# The Birth Prevalence of Spinal Muscular Atrophy: A Population Specific Approach in Estonia

**DOI:** 10.3389/fgene.2021.796862

**Published:** 2021-12-22

**Authors:** Siiri Sarv, Tiina Kahre, Eve Vaidla, Sander Pajusalu, Kai Muru, Haide Põder, Katrin Gross-Paju, Sandra Ütt, Riina Žordania, Inga Talvik, Eve Õiglane-Shlik, Kristina Muhu, Katrin Õunap

**Affiliations:** ^1^ Department of Clinical Genetics, Institute of Clinical Medicine, University of Tartu, Tartu, Estonia; ^2^ Department of Clinical Genetics, United Laboratories, Tartu University Hospital, Tartu, Estonia; ^3^ Tallinn Children’s Hospital, Tallinn, Estonia; ^4^ Centre for Neurological Diseases, West-Tallinn Central Hospital, Tallinn, Estonia; ^5^ Department of Health Technologies, eMed Lab, TalTech, Tallinn, Estonia; ^6^ Children’s Clinic, Institute of Clinical Medicine, University of Tartu, Tartu, Estonia; ^7^ Children’s Clinic, Tartu University Hospital, Tartu, Estonia

**Keywords:** spinal muscular atrophy, epidemiology, newborn screening, birth prevalence, neuromuscular disease

## Abstract

**Background:** Rare diseases are an important population health issue and many promising therapies have been developed in recent years. In light of novel genetic treatments expected to significantly improve spinal muscular atrophy (SMA) patients’ quality of life and the urgent need for SMA newborn screening (NBS), new epidemiological data were needed to implement SMA NBS in Estonia.

**Objective:** We aimed to describe the birth prevalence of SMA in the years 1996–2020 and to compare the results with previously published data.

**Methods:** We retrospectively analyzed clinical and laboratory data of SMA patients referred to the Department of Clinical Genetics of Tartu University Hospital and its branch in Tallinn.

**Results:** Fifty-seven patients were molecularly diagnosed with SMA. SMA birth prevalence was 1 per 8,286 (95% CI 1 per 6,130–11,494) in Estonia. Patients were classified as SMA type 0 (1.8%), SMA I (43.9%), SMA II (22.8%), SMA III (29.8%), and SMA IV (1.8%). Two patients were compound heterozygotes with an *SMN1* deletion *in trans* with a novel single nucleotide variant NM_000344.3:c.410dup, p.(Asn137Lysfs*11). *SMN2* copy number was assessed in 51 patients.

**Conclusion:** In Estonia, the birth prevalence of SMA is similar to the median birth prevalence in Europe. This study gathered valuable information on the current epidemiology of SMA, which can guide the implementation of spinal muscular atrophy to the newborn screening program in Estonia.

## Introduction

Spinal muscular atrophy (SMA) is an autosomal recessive neuromuscular disorder affecting approximately 1:10,000 live births, with a reported carrier frequency of 1:41 in Europe and 1:51 worldwide (Verhaart et al., 2017a; Wirth et al., 2020). SMA is commonly caused by large deletions or, more rarely, pathogenic variants affecting the survival of motor neuron 1 gene (*SMN1*) (Lefebvre et al., 1995; Finkel et al., 2018; Mercuri et al., 2018). The survival of the motor neuron 2 gene (*SMN2*) also produces SMN protein, although far less efficiently. Increased *SMN2* copies result in higher SMN protein levels, leading to a milder phenotype (Mailman et al., 2002; Wirth et al., 2006; Verhaart et al., 2017b; Calucho et al., 2018). Patients are classified into subtypes 0–IV according to age at the disease onset and achieved motoric abilities (Finkel et al., 2015; Wirth et al., 2020). The US Food and Drug Administration (FDA) and European Medicines Agency (EMA) have approved three treatment options for SMA, one gene replacement therapy (omasemnogene abeparvovec) and two *SMN2* splicing modifiers (nusinersen and risdiplam) (Messina and Sframeli, 2020; Wirth et al., 2020; Dimitrova, 2021). SMA is already added or is moving to the list of newborn screening (NBS) in many countries worldwide (Dangouloff et al., 2021). We aimed to investigate the birth prevalence of SMA to guide the implementation of SMA NBS soon in Estonia. We also review the literature about the current state of SMA NBS and describe a novel *SMN1* pathogenic point variant. This paper serves as an example of possibilities to improve treatment of rare diseases due to genomic developments.

## Materials and Methods

### Patients

Patients with a clinical suspicion of SMA were investigated and genotyped in the Department of Clinical Genetics of Tartu University Hospital. We retrospectively reviewed the clinical and laboratory data of all genetically diagnosed SMA patients in Estonia. We excluded two prenatally diagnosed and terminated cases. We created a registry based on case histories, including the data about date of birth, sex, age at the molecularly confirmed diagnosis, laboratory findings, and clinical SMA type.

### Methods

In Estonia, molecular analysis for SMA has been available since 1997 and performed to detect the homozygous deletion of *SMN1* gene exons 7 and 8. Restriction enzyme analysis was performed with enzymes *HinfI* for exon 7 and *DdeI* for exon 8. The products were analyzed using 1.5% agarose gel electrophoresis. The protocol was modified from the previously published method (Matthijs et al., 1996; Wirth et al., 1999).

Multiplex ligation-dependent probe amplification (MLPA) analysis has been available in Estonia since 2008. MLPA analysis using SALSA MLPA probemix P021-A1 SMA (MRC-Holland, Inc., Amsterdam, Netherlands) was carried out to detect the copy-numbers of exons 7 and 8 of *SMN1* and *SMN2* genes according to the manufacturer’s instructions. PCR products were analyzed on a capillary sequencer using the Genescan software (ABI 3130XL Genetic Analyzer; Applied Biosystems, Darmstadt, Germany). Coffalyser.NET software (MRC-Holland) was used for fragment analysis.

To detect point variants in patients with heterozygous deletion of *SMN1* gene and suspicion of SMA, we used TruSight One (TSO) and TruSight One Expanded (TSOE) panels (Illumina Inc., San Diego, California). These panels cover ∼4,800 and ∼6,700 genes associated with known genetic disorders or clinical phenotypes. A detailed description of the method was published earlier (Pajusalu et al., 2018). Detected variants were validated by Sanger sequencing.

Although most of the SMA literature uses the term “incidence”, we used the term “birth prevalence” because the genotype is present at birth. The literature on the birth prevalence of SMA was searched for through PubMed and references in the publications. We only included the publications in which the diagnosis of SMA was genetically confirmed.

### Statistical Analysis

We defined SMA birth prevalence as the number of SMA patients born each year divided by the recorded number of live births for the same year. Annual live-birth data were obtained from the database of Statistics Estonia. The total population of Estonia on December 31, 2020, was 1,329,460. There were 347,993 live births between the years 1996 and 2020 (www.stat.ee).

Live birth prevalence was estimated *via* the Generalized Linear Model Analysis using R version 4.0.2 (“R Core Team, 2020”, 2020). Poisson distribution was assumed for the prevalence cases. The default logarithmic link function was used and the only variable in the model was the observation year. The mean (expected) prevalence rate for a given year and the corresponding 95% confidence limits were predicted with R.

### Statement of Ethics

The study was approved by the Research Ethics Committee of the University of Tartu (259/T-2, 16.05.2016 263/M-16, 17.10.2016 and 278/T-19, 19.02.2018).

## Results

During a period of 24 years, from January 1997 to December 2020, a total of 548 *SMN* gene analyses were performed. The molecular diagnosis of SMA was confirmed in 57 patients (32 male, 25 female), including four sibling pairs. Thus the disease was diagnosed in 53 unrelated families. One patient was classified as SMA type 0 (1.8%), 25 as SMA I (43.9%), 13 as SMA II (22.8%), 17 as SMA III (29.8%), and one as SMA IV (1.8%).

Homozygous deletion of *SMN1* was detected in 55 (96.5%) patients, and two (3.5%) were compound heterozygotes of *SMN1* deletion and a point variant. Of the 55 SMA patients, 45 (78.9%) had a deletion of exons 7 and 8, and 10 (17.5%) had a deletion of only exon 7, which could be due to gene conversion. Most (7/10) of the patients with only *SMN1* exon 7 deletions were classified as SMA type III. The number of patients according to the SMA types and results of the gene analysis are given in Table 1.

**TABLE 1:** The number of patients according to the SMA subtypes and results of gene analysis.

SMA type	No. of Patients	Sex M/F	57 SMA patients with *SMN1* gene copy number		51 SMA patients with *SMN2* gene copy number^a^			
			0 copies	1 copy	1 copy	2 copies	3 copies	4 copies
0	1	1/-	1	—	1	-	-	—
I	25	13/12	23	2	1	19	1	—
II	13	7/6	13	—	—	2	10	—
III	17	10/7	17	—	—	2	8	6
IV	1	1/-	1	—	—	—	—	1

Abbreviations: F = Female, M = Male.

a The *SMN2* copy number was identified in 51 out of 57 patients.

Two patients were compound heterozygotes for a deletion of the whole *SMN1* gene (including *NAIP* and *GTF2H2* genes) and an intragenic variant NM_000344.3:c.410dup, p.(Asn137Lysfs*11). The novel *SMN1* variant c.410dup, p.(Asn137Lysfs*11), identified in exon 4, was absent from the Human Gene Mutation Database professional (HGMD pro), ClinVar, and gnomAD databases. Additionally, we did not find this variant in any publication other than the one from our group describing one of the patients (Puusepp et al., 2018). Both patients were classified as SMA type I and had two copies of *SMN2* (Table 2). Although they shared the same rare variant, we could not find a relationship based on the family history, at least not in the last 4–5 generations.

**TABLE 2:** Demographic and clinical data of patients heterozygous for point alterations.

Patient	SMA type	Sex	Age of onset	Age	Genetic variant (NM 000344.3)	*SMN2* /*NAIP* copies	Comorbidities
Case 1 (E01002376)	I	Female	4 days (reduced fetal movements, stimulated birth)	Died at 4 months	c.410dup p.(Asn137Lysfs*11)	2/1	patent ductus arteriosus, patent foramen ovale; tube feeding at the age of 1 month
Case 2 (E02178644)	I	Female	2 weeks	14 months	c.410dup p.(Asn137Lysfs*11)	2/1	Acute bronchiolitis, Guillain–Barré syndrome

**FIGURE 1:**
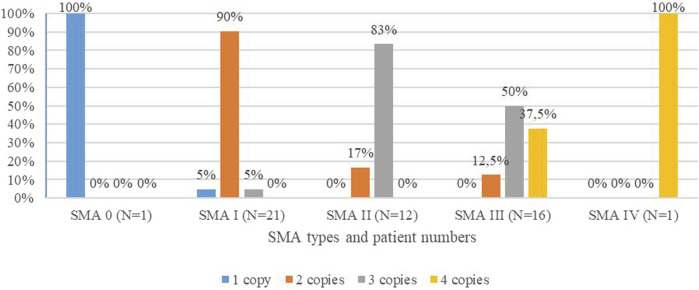
Distribution of *SMN2* copies in percentiles among SMA types of the studied cohort.

The *SMN2* copy number was identified in 51/57 patients. For six patients, DNA was unavailable because the patient was deceased before the MLPA test was available, and no DNA was stored. SMA type I patients carried one (5%), two (90%), or three (5%) copies of *SMN2*; SMA II patients had two (17%) or three (83%) copies of *SMN2*; and SMA III patients carried two (12.5%), three (50%), or four (37.5%) copies of *SMN2*. SMA type 0 and IV patients had one and four copies of *SMN2*, respectively, as shown in Figure 1 and Table 1 (Figure 1 and Table 1).

The mean age (shown with standard deviation) of confirmed genetic diagnosis of SMA was 0.4 months for SMA type 0; 3.2 ± 2.4 months (range 0.1–8 months) for SMA I; 18.8 ± 6.7 months (range 11–35 months) for SMA II; 13.4 ± 15.2 years (range 2–54 years) for SMA III; and 43 years for SMA IV. We excluded two patients with SMA type I whose molecular testing was performed postmortem as their parents asked for confirmation of the diagnosis and possibilities for prenatal diagnostics. Also, from the SMA type II group, three exceptions were made, as SMA was only clinically diagnosed in the period when the genetic basis of the disease was not yet known.

**FIGURE 2:**
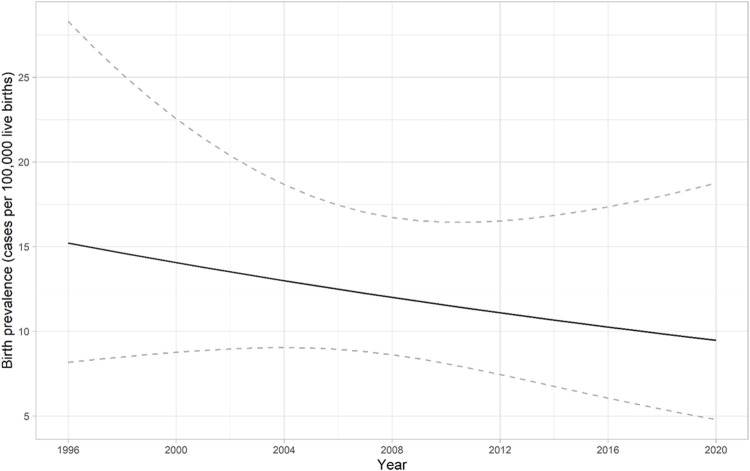
Birth prevalence of spinal muscular atrophy (SMA) in Estonia from 1996 to 2020. The solid line presents the predicted birth prevalence (number of cases among 100,000 live births). The dashed lines correspond to the 95% confidence interval for the birth prevalence. There was no statistically significant change in the live birth prevalence of SMA during this periood (*p* = 0.417).

SMA birth prevalence was determined using data from 42 SMA patients born during the years 1996–2020. One patient was diagnosed with SMA type 0 (2.4%), 24 patients with SMA I (57.1%), 9 patients with SMA II (21.4%), and 8 patients with SMA III (19.1%). The birth prevalence of SMA in Estonia is 1 per 8,286 (95% CI 1 per 6,130–11,494) live births (12.1 per 100,000 live births) (Figure 2; Table 3). There was no statistically significant change in the live birth prevalence of SMA during this periood (*p* = 0.417).

**TABLE 3:** Studies of birth prevalence of SMA in different countries based on genetic testing.

Country	Timeframe	SMA				References
		Cases	Study population	Per 100,000	Birth prevalence	
Estonia	1994–2003	15	129,832	11.6^a^	1:8,655	Vaidla et al. (2006)
	01.1996–12.2020	42	347,993	12.1^a^	1:8,286	This study
Sweden (Western)	1980–2006	45	531,746	8.5^a^ ^,^ ^b^	1:11,817	Arkblad et al. (2009)
Poland	1998–2005	304	2,963,783	10.3^a^	1:9,749	Jedrzejowska et al. (2010)
United States (Ohio)	–	4	40,103	10.0^c^	1:10,026	Prior et al. (2010)
Europe	2011–2015	3,776	22,325,221	11.9^d^ ^,^ ^e^ (range 6.3–26.7)	1:8,403 (range 1:3,900–16,000)	Verhaart et al. (2017a)
Taiwan	11.2014–09.2016	7	120,267	5.8^c^	1:17,181	Chien et al. (2017)
	*–01.2021*	20	419,102	4.8	1:20,955	Dangouloff et al. (2021)
United States (NYC/NYS)	01.2016–01.2017	1	3,826	26.1^c^	1:3,826	Kraszewski et al. (2018)
	10.2018–10.2019 (–02.2020)	8 (15)	225,093 (∼314,000)	3.6^f^ (∼4.8)	1:28,137 (∼1:20,933)	Kay et al. (2020)
	*–01.2021*	180	2,395,718	*7.5*	1:13,310	Dangouloff et al. (2021)
Greece	1995–2018	∼200	2,437,348	∼8.2^a^	∼1:12,000	Kekou et al. (2020)
Japan	01.2018–04.2019	0	4,157	0^c^	0	Shinohara et al. (2019)
	*–01.2021*	0	22,209	0	0	Dangouloff et al. (2021)
Germany	2014	97	714,927	13.6^a^	1:7,370	König et al. (2019)
Germany	01.2018–02.2019	22	165,525	13.3^c^	1:7,524	Vill et al. (2019)
	–08.2019	30	213,279	14^c^	1:7,109	Czibere et al. (2020)
	–11.2019	38	278,970	13.6^c^	1:7,350	Müller-Felber et al. (2020)
	–01.2020	43	297,163	14.5^c^	1:6,910	Vill et al. (2021)
Belgium	03.2018–06.2019	5	35,000	14.3^c^	1:7,000	(Boemer et al., 2019b; 2019a)
	*–01.2021*	9	127,329	7.1^c^	1:14,148	Dangouloff et al. (2021)
Australia	08.2018– 07.2019	9	103,903	8.7^c^	1:11,545	Kariyawasam et al. (2020)
	*–01.2021*	19	202,388	9.4	1:10,652	Dangouloff et al. (2021)
Italy	*09.2019–01.2021*	12	58,558	20.5^c^	1:4,880	Dangouloff et al. (2021)
Russia	*08.2019–01.2021*	0	12,000	0	0	Dangouloff et al. (2021)
Canada	01.2020–	—	—	—	—	McMillan et al. (2021)
	*–01.2021*	5	139,810	3.6	1:27,962	Dangouloff et al. (2021)

a A retrospective study.

b Patients were younger than 16 years of age.

c A pilot study of newborn screening of Spinal muscular atrophy.

d Median birth prevalence of 18 countries in Europe.

e Combined data from the TREATNMD Global SMA Patient Registry and the Care and Trial Sites Registry (CTSR).

f Official newborn screening of Spinal muscular atrophy.

In italic is the latest information about SMA NBS by countries from an article by (Dangouloff et al., 2021).

## Discussion

During study period, we detected 57 patients with SMA, most of them had a homozygous deletion of *SMN1* exons 7 and 8 (Table 1). Most of the SMA patients were classified as SMA type I (43.9%), followed by SMA III (29.8%) and SMA II (22.8%), similar to a previous report from European populations (Verhaart et al., 2017b).

We performed the MLPA analysis in 51 out of 57 SMA patients to detect the copy number of the *SMN2* gene, which plays an important role in the disease’s severity (Mailman et al., 2002; Wirth et al., 2006; Verhaart et al., 2017a; Calucho et al., 2018; Wirth et al., 2006). SMA type 0 patient carried one copy of *SMN2*, which is usual to SMA 0 (Finkel et al., 2015; Grotto et al., 2016). Our results (Figure 1; Table 1) correlate with the published data, in which 73% of SMA type I patients carry two *SMN2* copies, 78% of SMA II patients carry three copies, 49 and 44% of SMA III patients carry three and four copies, respectively, and 75% of SMA IV patients have four *SMN2* copies (Calucho et al., 2018).

In our study cohort, 4% of patients have one copy, 45% have two copies, 37% have three copies, and 14% have four copies of *SMN2*, similar to a study from Spain and an NBS study from Germany (Calucho et al., 2018; Vill et al., 2021). Although other factors are involved with the disease severity (Wirth et al., 1999; Prior et al., 2009; Bernal et al., 2010), the *SMN2* copy number is a major modifier and is important for prognosis and therapeutic approaches. It is recommended by the SMA NBS Multidisciplinary Working Group that patients with one to four copies of *SMN2* need immediate treatment, and a strategy of watchful waiting is applied for those with five *SMN2* copies (Glascock et al., 2020).

Most commonly, 96% of SMA patients carry a homozygous deletion of *SMN1* exon 7 and 8 or exon 7 only (Kolb and Kissel, 2015; Verhaart et al., 2017a; Wirth, 2000); the remaining 4% are compound heterozygotes (Mercuri et al., 2018; Wirth et al., 1999). In our study, the homozygous deletion of *SMN1* was detected in 55 (96.5%) patients, and 2 (3.5%) patients were compound heterozygotes (Table 1). Recurrent point variants in the compound heterozygous patients are missense or nonsense and located in exons 3 and 6 (Alías et al., 2009; Mendonça et al., 2020). Currently, the HGMD-Pro database shows 123 different pathogenic variants described across *SMN1*: 48 missense/nonsense, 25 gross deletions, 21 small deletions, 17 small insertions/duplications, and 13 splicing alterations (Stenson et al., 2020). In the ClinVar database, there are 50 pathogenic variants reported in the *SMN1* gene, 21 single nucleotide substitutions, 19 deletions, 9 duplications, and 4 small insertions (https://www.ncbi.nlm.nih.gov/clinvar).

The identified *SMN1* exon 4 variant NM_000344.3:c.410dup, p.(Asn137Lysfs*11) in our study is novel, only published by our group (Puusepp et al., 2018). Pathogenicity was classified by the American College of Medical Genetics and Genomics (ACMG) variant interpretation guidelines (Richards et al., 2015). By ACMG guidelines, the variant is pathogenic because it is absent in population databases, predicted a null variant, and was detected *in trans* with the deletion of *SMN1* exons 7 and 8 (Puusepp et al., 2018). Both compound heterozygous patients with *SMN1* point variant were female, classified as SMA type I, and had two copies of *SMN2*. Since patients with two copies of *SMN2* most often are classified as SMA type I (Calucho et al., 2018), it is unclear whether this rare variant is linked to a more severe phenotype. Also, the course of the disease in these patients was different (Table 2). In case 1, the symptoms started on the fourth day of life, but decreased fetal movements were observed before birth. She was clinically diagnosed early, but molecularly confirmed only 11 years later. Her disease progressed quickly, and she died at the age of 4 months. In case 2, clinical symptoms emerged later with breathing difficulties, and from 1.5 months of age, the muscle weakness rapidly progressed. At first, case 2 was diagnosed with Guillain-Barré syndrome, but a suspicion of SMA lead to a molecularly confirmed diagnosis at the age of 3 months and prompted immediate treatment with risdiplam. At the age of 14 months, case 2 is still alive.

The mean age (with standard deviation) of confirmed genetic diagnosis of SMA in Estonia was 3.2 ± 2.4 months for SMA type I; 18.8 ± 6.7 months for SMA II and 13.4 ± 15.2 years for SMA III. In the study of Lin et al., the weighted mean ages of confirmed SMA genetic diagnosis were 6.3 ± 2.2 months for SMA type I; 20.7 ± 2.6 months for SMA II; and 4.2 ± 1.1 years for SMA III (Lin et al., 2015). A study by Pera et al. showed the mean age of confirmed genetic diagnosis to be 4.7 ± 2.8 months in SMA type I; 15.6 ± 5.9 months in SMA II; and 4.34 ± 4.01 years in SMA III (Pera et al., 2020). Our study’s faster diagnosis of SMA type I could be due to the centralized molecular diagnostic service in Estonia, where all the patients’ samples are tested and neonatologists and child neurologists are accessible. Delayed molecular diagnosis of SMA type III in Estonia could be explained by different reasons: patients were born before the SMA genetic test was available, symptoms were milder and therefore less specific, or patients were diagnosed clinically before and molecularly tested years later to confirm the diagnosis.

In Estonia, the birth prevalence of genetically confirmed SMA has previously been reported in a smaller cohort (15 patients) between the years 1994–2003 as 1:8,655 (Vaidla et al., 2006). The present study shows similar results (1:8,286), which indicates that our diagnostic efficacy has stayed on the same level. Due to that, we think that no SMA patient born in the last 25 years has been missed. However, there might be a higher rate of late-onset SMA type III and type IV patients in the future, and asymptomatic patients may be increasingly diagnosed (Prior et al., 2004; Jędrzejowska et al., 2008).

There are no published data about SMA birth prevalence from the nearby countries (e.g., Latvia, Lithuania, and Finland), although on December 1, 2020, Latvia started a yearlong SMA pilot NBS project (“Pilot study”, RSU, 2020). The closest data originate from Sweden, with a birth prevalence of 1:11,817. Similar to our findings, the birth prevalence in Poland was 1:9,749; European combined data 1:8,403 (11.9 per 100,000) and SMA NBS pilot results from Germany 1:6,910; Belgium 1:7,000 (*1:14,148*); and Australia 1:11,545 (*1:10,652*). Similar retrospective studies have been performed in Germany and Greece, and their birth prevalence was 1:7,370 and 1:12,187, respectively, which is comparable to our results. Also, a lower birth prevalence was derived from SMA NBS studies in the United States of 1:28,137 (*1:13*,*310*) and Taiwan 1:17,181 (*1:20*,*955*) (Table 3).

Newborn screening for SMA identifies affected infants before the clinical onset. It allows early treatment to avoid the motor neurons’ degradation since the death of spinal motor neurons causes the rapid onset of muscle weakness in patients. Globally, SMA NBS has been implemented in nine countries, including three in Europe: Germany, Belgium, and Italy (Table 3). Spain, the United Kingdom, France, Poland, Norway, Denmark, Slovakia, Slovenia, Serbia, the Netherlands and many other countries in Europe also plan to implement SMA screening soon (Loeber et al., 2021; SMA Newborn Screening Alliance, 2021). Most SMA NBS programs use qPCR of DNA extracted from dry blood spots (DBS) to detect the homozygous deletion of *SMN1* exon 7. To confirm the deletion and the *SMN2* copy number, they use the MLPA test performed from a secondary blood sample. At the moment, 288 newborns with SMA have been detected out of 3,674,277 newborns screened, with a birth prevalence of 1:12,757 (Dangouloff et al., 2021). In Germany and the United States, the SMA patients found by NBS with two or three copies of *SMN2* who were asymptomatic before the nusinersen or onasemnogene abeparvovec treatment were also symptom-free at their last follow-up (Kay et al., 2020; Vill et al., 2021). This finding correlates with previous clinical studies performed with nusinersen and onasemnogene abeparvovec-xioi (De Vivo et al., 2019; Hoy, 2019; Stevens et al., 2020; Wirth et al., 2020). Thus, SMA NBS has shown real benefits during the short time it has been implemented. The main advantage would be that *SMN1*-deleted newborns treated before the onset of symptoms will develop almost as well as healthy newborns (Dangouloff and Servais, 2019; De Vivo et al., 2019). Also, the testing has avoided diagnostic delay and helped to identify other family members who have SMA or are the carriers and at risk of having affected offspring (Aharoni et al., 2020).

The carrier screening could also be beneficial as an alternative to SMA NBS, since the long term effect of the treatment is not yet known, especially when it is performed using NGS, as it could detect point variants that SMA NBS would miss (Prior et al., 2010; Sugarman et al., 2012; Verhaart et al., 2017b; Wijaya et al., 2020). In Estonia carrier screening programs could happen in collaboration of Estonian Genome Center where population-based biobank of Estonia has been established with the data of 200,000 Estonian residents which reflects almost 20% of the adult Estonia`s population (“Estonian Biobank”, Genomics, 2018). New research program could be started to offer SMA carrier testing for partners of Estonian biobank donors who have been identified as SMA carriers. There are only few national adult carrier screening programs carried out in the whole world (Aharoni et al., 2020; Zhang et al., 2020; Nilay et al., 2021). However, Israel study showed that SMA carrier screening has been effective in increasing prenatal detection of SMA but has had no effect on the rate of confirmed postnatal diagnoses (Aharoni et al., 2020).

The European Alliance for NBS in SMA requests that by 2025, all European countries with NBS programs should include a test for SMA for all newborn children. In a global overview, the countries without an implemented SMA NBS place high importance on implementing SMA NBS, and 37 out of 76 countries reported plans for adding spinal muscular atrophy to the NBS program (Dangouloff et al., 2021). At present, the newborns are screened for 21 conditions in Estonia, compared to Latvia, Lithuania, and Finland, 6, 4, and 22 conditions, respectively (Loeber et al., 2021). Pilot program to implement the SMA NBS will be started from year 2022 in Estonia led by our working group.

## Conclusion

SMA diagnostics have been effective in Estonia, as the birth prevalence of SMA is similar to the median birth prevalence in Europe. This valuable information gives us an effective starting point for the implementation of SMA to the newborn screening program in Estonia.

## Data Availability

The data analyzed in this study is subject to the following licenses/restrictions: we analysed molecular results performed in Tartu University Hospital and these data are not publicly available. Requests to access these datasets should be directed to Katrin Õunap, email: katrin.ounap@kliinikum.ee.
